# Reviewer acknowledgement 2025

**DOI:** 10.3322/caac.70033

**Published:** 2025-09-15

In order to maintain the high standards of *CA*’s content, the Editors of *CA* rely on the knowledge and dedication of many experts in deciding which topics to pursue, which manuscripts to publish, and what modifications to make to ensure medical and scientific accuracy and suitability for our readers. We thank our Associate Editors and our Editorial Advisory Board, who continue to provide these services for us time and time again.

We are also greatly indebted to the effort and expertise of the following individuals for reviewing manuscripts for the journal from July 1, 2024, to June 30, 2025. These individuals go beyond expectations by consistently and expeditiously delivering comprehensive, discerning reviews.

Lauren Antrim

Pedro Barata

Nabila Bennani

Ari Birnbaum

Sue Bornstein

Donald Cannon

Michael Carney

Elizabeth Cathcart‐Rake

Annie Chan

Huizi Chen

Carissa Chu

Sean David

Ruth Etzioni

Robert Ferris

Courtney Finlayson

Bryan Fuchs

Elizabeth Garrett‐Mayer

William Hall

Michael Halpern

Ole‐Petter Hamnvik

David Hui

Linda Jacobs

Salvador Jaime‐Casas

Rebecca Johnson

Corinne Joshu

David Keefe

WonSeog Kim

Elise Kohn

Lindsay M. Kuroki

Rita Kuwahara

Richard Lee

Shing Lee

Phebe Lemert

Stanley Liauw

Stephen Liu

Shail Maingi

Sendurai Mani

Jonathan Marron

Brittany C. McGill

Jacob Miller

Paul Montgomery

Susan O'Brien

Krishnan Patel

Rodolfo Alberto Rey

Tina Rizack

Nabil Saba

Stephanie Smith

Umang Swami

Wade Swenson

Russell Taichman

Yungan Tao

Molly Taylor

Ayalew Tefferi

Sarah Temkin

William Tew

Premal Thaker

Jonathan Thompson

Michael Thun

Katherine Tossas

Zaza Tsitsishvili

Adam Wahida

Thomas Zilli

Miguel Zugman

